# Resting heart rate and incident venous thromboembolism: the Multi-Ethnic Study of Atherosclerosis

**DOI:** 10.1136/openhrt-2019-001080

**Published:** 2020-02-20

**Authors:** Josephine Awotoye, Oluwaseun E Fashanu, Pamela L Lutsey, Di Zhao, Wesley T O'Neal, Erin D Michos

**Affiliations:** 1 Department of Biology, University of Minnesota, Minneapolis, Minnesota, USA; 2 Department of Medicine, Saint Agnes Hospital, Baltimore, Maryland, USA; 3 The Ciccarone Center for the Prevention of Cardiovascular Disease, Johns Hopkins School of Medicine, Baltimore, Maryland, USA; 4 Division of Epidemiology and Community Health, School of Public Health, University of Minnesota, Minneapolis, Minnesota, USA; 5 Department of Epidemiology, Johns Hopkins Bloomberg School of Public Health, Baltimore, Maryland, USA; 6 Department of Medicine, Division of Cardiology, Emory University School of Medicine, Atlanta, Georgia, USA

**Keywords:** epidemiology, deep vein thrombosis, venous thromboembol, heart rate variability

## Abstract

**Objective:**

Venous thromboembolism (VTE) is associated with significant morbidity and mortality. Resting heart rate (RHR), which may be modifiable through lifestyle changes, has been shown to be associated with cardiovascular disease risk and with inflammatory markers that have been predictive of VTE incidence.

**Methods:**

We examined whether RHR is also associated with VTE incidence independent of these risk factors. We studied 6479 Multi-Ethnic Study of Atherosclerosis participants free from clinical VTE at baseline who had baseline RHR ascertained by 12-lead ECG. VTE events were recorded from hospital records and death certificates using International Classification of Diseases (ICD)-9 and ICD-10 codes. We categorised RHR as <60, 60–69, 70–79 and ≥80 bpm. We used Cox hazard models to determine the association of incident VTE by RHR.

**Results:**

Participants had mean (SD) age of 62 (10) years and RHR of 63 (10) bpm. RHR was cross-sectionally correlated with multiple inflammatory and coagulation factors. There were 236 VTE cases after a median follow-up of 14 years. Compared with those with RHR<60 bpm, the HR (95% CI) for incident VTE for RHR≥80 bpm was 2.08 (1.31 to 3.30), after adjusting for demographics, physical activity, smoking, diabetes and use of atrioventricular (AV)-nodal blockers, aspirin and anticoagulants, and remained significant after further adjustment for inflammatory markers (2.05 (1.29 to 3.26)). Results were similar after excluding those taking AV-nodal blocker medications. There was no effect modification of these associations by sex or age.

**Conclusion:**

Elevated RHR was positively associated with VTE incidence after a median of 14 years; this association was independent of several traditional VTE and inflammatory markers.

**Key questions**

**What is already known about this subject?**

- An elevated resting heart rate (RHR) may reflect decreased parasympathetic tone and increased sympathetic stimulation and is associated with increased cardiovascular disease (CVD) risk. Elevated RHR has been associated with inflammatory markers, and alteration in the sympathetic nervous system may predispose to thrombosis. We examined whether RHR was associated with increased incidence of venous thromboembolism (VTE).

**What does this study add?**

- In a large diverse community cohort of individuals free of VTE, CVD, atrial fibrillation and active cancer at baseline, we demonstrated that RHR was significantly associated with a twofold increased risk of incidence of VTE over a median of 14 years. This association was independent of several demographics, lifestyle and inflammatory factors. RHR also had a significant and positive correlation, though weak, with several inflammatory and coagulation factors cross-sectionally.

**How might this impact on clinical practice?**

- RHR can readily be measured at clinic visits or through personal fitness trackers; however, it remains underused for the purpose of cardiovascular and thrombosis risk assessments. Furthermore, RHR may be modifiable with improvement in fitness or with pharmacotherapy. Further studies are needed to determine whether the associations of RHR and VTE are causal or secondary to another underlying process, and whether modification of RHR can reduce VTE risk.

## Introduction

Venous thromboembolism (VTE) may present as a deep venous thrombosis (DVT) and/or a pulmonary embolism (PE)^1^ and is associated with significant morbidity and mortality.^2 3^ Although both diseases (PE and DVT) have different clinical manifestations, they represent the same disease process and share similar risk factors.^4 5^ The identification of modifiable risk factors is paramount to reducing the disease incidence and prevalence.^1^ Resting heart rate (RHR) is an easily assessable clinical variable that is potentially modifiable with lifestyle changes and/or pharmacotherapy. In the previous studies, a higher RHR has been shown to be a significant predictor of all-cause mortality and cardiovascular disease (CVD), independent of traditional risk factors.^6–10^ The association between RHR and CVD also differed by sex.^10^ Compared with women, men had a lower average RHR but a greater CVD risk associated with elevated RHR.^10^ However, whether RHR is associated with VTE, specifically, is unknown. RHR has also been shown to be associated with several inflammatory markers,^7 11^ which in turn have been predictive of VTE incidence.^12 13^ A prior study also showed a positive and significant correlation between RHR and several coagulation factors.^14^ Taken together, a higher RHR may predispose to a hypercoagulative state, which may lead to a VTE event. However, to the best of our knowledge, at the time we initiated this work, no epidemiological study had yet to evaluate the association between RHR and VTE incidence in a general community-based cohort. Therefore, the aim of our study is to determine the prospective association between RHR and incidence of VTE among participants of the Multi-Ethnic Study of Atherosclerosis (MESA), who were free of clinical CVD and VTE at baseline and whether this association differs by sex.

## Methods

The MESA population consists of 6814 men and women who, at recruitment in 2000–2002, were aged 45–84 years and without clinical atherosclerotic CVD, heart failure or atrial fibrillation, and were not undergoing active treatment for cancer.^15^ Participants were of four races/ethnicities including white, black, Hispanic and Chinese, and recruited from six communities in the USA including Los Angeles County, California; Chicago, Illinois; Baltimore/Baltimore County, Maryland; Saint Paul, Minnesota; Forsyth County, North California and Northern Manhattan/Bronx, New York. After baseline (2000–2002), participants attended up to five additional clinic visits at exam 2 (2002–2004), exam 3 (2004–2005), exam 4 (2005–2007), exam 5 (2010–2012) and exam 6 (2016–2018). Additionally, they took part in regular phone calls which queried recent hospitalisations. The design of the MESA study has been previously published.^15^

After exclusions for missing baseline RHR (n=48), missing covariates in our primary model (n=258) or missing VTE follow-up data (n=29), a total of 6479 participants were included for our primary analysis for incident VTE (online supplementary figure 1). In a supplemental analysis conducted to help establish potential mechanisms, we also examined the cross-sectional association of RHR with several plasma haemostatic factors and endothelial markers (further described below in this section). Some of these biomarkers were measured in the entire cohort, while others were measured in a random sample of a 1000 MESA participants.^16^ The baseline characteristics of these ‘MESA 1000’ participants did not differ from that of the entire MESA cohort.

10.1136/openhrt-2019-001080.supp1 Supplementary data

For the primary analysis, we used data regarding RHR and clinical and laboratory covariates that were obtained at the MESA baseline examination. Participants were instructed to fast for at least 12 hours and avoid heavy exercise before the exam. A 12-lead ECG obtained at rest was used to record the RHR of participants at baseline.

Surveys and questionnaires were used to gather information on age, sex, race/ethnicity, education and smoking status. Information on physical activity was obtained by asking questions on time and frequency of physical activity during the week using a survey instrument adapted from the Cross-Cultural Activity Participation Study,^17^ and total minutes of moderate and/or vigorous exercise per week were calculated. Body mass index (BMI) was measured as the weight (kilograms)/height (kg/m^2^). Diabetes was based on self-report, medication history as well as a fasting glucose level of ≥126 mg/dL. A medication inventory approach^18^ was used to determine atrioventricular (AV)-nodal blocker use (defined as participants who were on either beta-blockers, verapamil or diltiazem), aspirin and oral anticoagulants, as well as use of antihypertensive and lipid-lowering therapies. The Chronic Kidney Disease Epidemiology equation was used to calculate the estimated glomerular filtration rate (eGFR).^19^

Serum concentration of high-sensitivity C reactive protein (hsCRP) and fibrinogen were measured using the Dade Behring Nephelometer II Analyzer System (BNII) (Deerfield, Illinois, USA).^11^ Interleukin-6 (IL-6) serum concentration was measured using ultrasensitive ELISA (Quantikine HS Human IL-6 Immunoassay, R&D Systems).^11^ D-dimer was measured using Sta-R analyzer (Liatest D-DI; Diagnostica Stago, Parsippany, New Jersey, USA).^20^

As previously described, plasma haemostatic factors and endothelial markers were measured in various samples of the MESA cohort.^16^ At baseline, in the entire cohort, factor VIII and plasmin–antiplasmin (PAP) were measured, whereas plasminogen activator inhibitor-1 (PAI-1), von Willebrand factor (VWF), soluble thrombomodulin (STM) and E-selectin were measured only in the ‘MESA 1000’ random sample.^16^ Intercellular adhesion molecule 1 (ICAM-1) was measured from the ‘MESA 1000’ sample and also from all participants enrolled before February 2003.^16^ Due to missing data and exclusions, the numbers included slightly varied across these biomarkers.

Participants were followed from study baseline (2000–2002) to 2015. They were contacted by telephone every 9–12 months to obtain information regarding self-reported hospitalisation, and the medical records from these hospitalisations were obtained for review. Incident VTE events were identified via these hospitalisation records and also from death certificates by using International Classification of Diseases (ICD)-9 and ICD-10 codes. These codes were predetermined by a panel of VTE experts in the MESA coordinating centre. They were chosen to be consistent with the ICD codes used for the Longitudinal Investigation of Thromboembolism (LITE) study^21^ whose goal was to determine the incidence of VTEs in two community-based cohorts: the Atherosclerosis Risk in Communities (ARIC)^22^ and the Cardiovascular Health Study cohorts^23^ (see online supplementary table 1 for the specific ICD-9 and ICD-10 codes used).

We stratified baseline characteristics by the presence/absence of incident VTE. We present continuous variables as mean±SD or median (25–75th percentile) for normally distributed and skewed variables, respectively. We present categorical variables as frequency (percentage). We used two samples t-test, Mann-Whitney test or X^2^ test to describe differences between groups as appropriate. We used multivariable-adjusted Cox proportional hazards regression models to determine the HRs and 95% CIs of incident VTE by RHR groups categorised by previously established cut points (<60, 60–69, 70–79 and ≥80 bpm).^10 24 25^ We tested for trends across RHR categories by using an ordinal variable for each RHR category and modelling this as a continuous variable for association with VTE. We also examined RHR as a continuous variable per 10 bpm increment. We tested non-violation of the Cox proportional assumption using Schoenfeld residuals.

We used progressively adjusted models. In model 1, we adjusted for demographic variables of age (continuous), sex (men; women) and race/ ethnicity (white; black; Hispanic; Chinese). Model 2 (our primary analytical model) was further adjusted for socioeconomic and lifestyle factors such as education (<high school; high school or vocational school; college, graduate or professional school), BMI (continuous) and physical activity level (metabolic equivalent of task (MET) minutes /week of moderate or vigorous activity; log-transformed continuous). Model 3 was additionally adjusted for cigarette smoking (current; former; never), diabetes (yes; no), eGFR (continuous), use of AV nodal blocking agents (yes; no), aspirin (yes; no) and anticoagulants (yes; no). Note that the use of oral anticoagulants was very low in MESA (0.4% of our analytic sample), as MESA excluded those with clinical CVD and atrial fibrillation at baseline. We did not adjust for participants with oral contraceptive or hormone therapy use as data were only limited to women and was not associated with VTE in this cohort (not shown). In a final model, model 4, we further adjusted for the inflammatory and coagulation markers (all log-transformed, continuous) of hsCRP, IL-6, fibrinogen and D-dimer, which may play an intermediary role in the relationship between RHR and VTE.

To further characterise the relationship between RHR and incident VTE, we used restricted cubic splines centred at the median RHR (62 bpm) with five knots placed at the 5th, 27.5th, 50th, 72.5th and 95th percentiles adjusted for variables in our main analytic model (model 2).

We performed several sensitivity analyses. First, we explored if the association between RHR and VTE differed by sex based on a priori hypotheses.^10^ We also examined interactions by age groups (per 10-year increments). Second, we excluded participants on AV-nodal blocker medications. Third, we modelled RHR and some covariates in our models as time-varying, with variables updated at MESA exams 2, 3, 4 and/or 5, as available (of note, since VTE events were recorded through 2015, data from MESA exam 6 which began in 2016 was not included). Lastly, to evaluate for potential mechanisms, we examined the partial correlations of RHR with various inflammatory and coagulation factors in MESA, using Spearman’s correlation (r), after adjusting for age, sex, and race/ethnicity.^16^ We considered a p<0.05 to be statistically significant, and performed our analyses using Stata V.15.

## Results

The mean (SD) age of participants (n=6479) was 62 (10) years and RHR of 63 (10) bpm; 53% were women, 39% white, 27% black, 22% Hispanic and 12% Chinese Americans. Participants who had VTE at follow-up had a significantly higher mean RHR, age, BMI and lower eGFR at baseline when compared with participants who did not develop a VTE. Participants who developed incident VTE also had higher median hsCRP, IL-6, fibrinogen and D-dimer levels and a higher proportion were using anticoagulants and aspirin at baseline. Sex, educational status, smoking status, physical activity, diabetes and use of AV-nodal medications at baseline were not statistically significantly related to incident VTE (table 1). The baseline characteristics according to RHR groups are presented in online supplementary table 2. Participants with a higher RHR were more likely to be women, have lower average physical activity, have a higher prevalence of diabetes and have higher levels of the inflammatory markers.

**Table 1:** Baseline characteristics of study participants by incident venous thromboembolism (VTE): the Multi-Ethnic Study of Atherosclerosis, 2000–2002*

Baseline characteristics	Total	No VTE	VTE	P value
N	6479	6243	236	–
Heart rate, bpm	63.1±9.6	63±9.6	64.6±11.1	0.01
Heart rate, bpm*	62 (56–69)	62 (56–69)	63 (56–73)	0.04
Age, years	62.1±10.2	62±10.2	66.6±9.4	<0.001
Women	3423 (52.8%)	3296 (52.8%)	127 (53.8%)	0.76
Race/ethnicity				<0.001
White	2504 (38.7%)	2403 (38.5%)	101 (42.8%)	–
Black	1757 (27.1%)	1668 (26.7%)	89 (37.7%)	–
Hispanic	1431 (22.1%)	1394 (22.3%)	37 (15.7%)	–
Chinese	787 (12.2%)	778 (12.5%)	9 (3.8%)	–
Education				0.69
Less than high school	1176 (18.2%)	1134 (18.2%)	42 (17.8%)	–
High school or vocational school	2683 (41.4%)	2579 (41.3%)	104 (44.1%)	–
College, graduate or professional school	2620 (40.4%)	2530 (40.5%)	90 (38.1%)	–
Smoking status				0.33
Never	3268 (50.4%)	3160 (50.6%)	108 (45.8%)	–
Former	2381 (36.8%)	2285 (36.6%)	96 (40.7%)	–
Current	830 (12.8%)	798 (12.8%)	32 (13.6%)	–
BMI, kg/m^2^	28.3±5.4	28.2±5.4	30.5±6.2	<0.001
Physical activity, MET-minutes/week†	4020 (1980–7515)	4035 (1980–7545)	3554 (1864–6544)	0.13
Diabetes	814 (12.6%)	777 (12.5%)	37 (15.7%)	0.14
eGFR, ml/min per 1.73 m^2^	77.7±16.2	77.9±16.1	73.5±18.4	<0.001
AV nodal blocker medication	829 (12.8%)	792 (12.7%)	37 (15.7%)	0.18
Anticoagulant use	24 (0.4%)	20 (0.3%)	4 (1.7%)	0.001
Aspirin use	1631 (25.2%)	1556 (24.9%)	75 (31.8%)	0.02
hsCRP, mg/L†	1.9 (0.8–4.2)	1.9 (0.8–4.2)	2.5 (1.2–4.5)	0.002
Interleukin-6, pg/mL†	1.2 (0.8–1.9)	1.2 (0.8–1.9)	1.4 (0.9–2.6)	<0.001
Fibrinogen, mg/dL†	337 (295–388)	337 (294–387)	346 (309–407)	0.01
D-dimer, μg/mL†	0.2 (0.1–0.4)	0.2 (0.1–0.4)	0.4 (0.2–0.6)	<0.001

*Data are presented as mean±SD for continuous variables and as frequency (percentage) for categorical variables, unless otherwise specified. P values derived from two samples t-test or Mann-Whitney test for continuous variables and X^2^ test for categorical variables.

†Data presented as median (25–75th percentile).

AV, atrioventricular; BMI, body mass index; bpm, beats per minute; eGFR, estimated glomerular filtration rate; hsCRP, high-sensitivity C reactive protein; MET, metabolic equivalent of task.

After a median of 14 years of follow-up, there were 236 incident cases of VTE. Of these cases, one event was a VTE mortality. The unadjusted VTE incidence rate (95% CI) in this population who were free from VTE at baseline was 2.93 (2.58 to 3.33) per 1000 person-years. The HRs (95% CIs) for incident VTE after accounting for baseline demographics (model 1) comparing the participants with RHR of 60–69 bpm, 70–79 bpm and ≥80 bpm to participants with RHR≤60 bpm was 0.92 (0.67 to 1.25), 1.47 (1.04 to 2.08) and 2.49 (1.60 to 3.88), respectively, (p for trend<0.001). Compared with RHR≤60 bpm, the association between elevated RHR≥80 bpm with VTE incidence remained statistically significant in our main model (model 2) which further accounted for socioeconomic and lifestyle factors (HR 2.01 (1.28, 3.15)) and did not differ significantly by sex (p for interaction=0.10) or by age groups (p for interaction=0.64, online supplementary table 3). In our fully adjusted model (model 4) which further accounted for inflammatory markers associated with VTE, the HR (95% CI) for incident VTE was 2.05 (1.29 to 3.26) for RHR≥80 bpm compared with ≤60 bpm. Results also remained statistically significant when we modelled RHR as a continuous variable in all models (table 2).

**Table 2:** Risk of incident venous thromboembolism (VTE) associated with resting heart rate: the Multi-Ethnic Study of Atherosclerosis, 2000–2015

Resting heart rate	≤60 bpm	60–69 bpm	70–79 bpm	≥80 bpm	P for trend	Per 10 bpm increment
N	2434	2540	1143	362	–	6479
VTE; n (%)	83 (3.4)	75 (3.0)	52 (4.6)	26 (7.2)	–	236 (3.6)
Person-years	30 559	31 820	14 013	4219	–	80 610
Incidence rate (95% CI)*	2.72 (2.19 to 3.37)	2.36 (1.88 to 2.96)	3.71 (2.83 to 4.87)	6.16 (4.20 to 9.05)	–	2.93 (2.58 to 3.33)
HRs (95% CI)†						
Model 1	1 (reference)	0.92 (0.67 to 1.25)	**1.47 (1.04 to 2.08)**	**2.49 (1.60 to 3.88)**	<0.001	**1.24 (1.09 to 1.41)**
Model 2	1 (reference)	0.88 (0.65 to 1.21)	1.31 (0.92 to 1.87)	**2.01 (1.28 to 3.15)**	0.004	**1.17 (1.02 to 1.33)**
Model 3	1 (reference)	0.88 (0.64 to 1.20)	1.33 (0.93 to 1.91)	**2.08 (1.31 to 3.30)**	0.004	**1.18 (1.03 to 1.35)**
Model 4	1 (reference)	0.90 (0.66 to 1.24)	1.37 (0.95 to 1.96)	**2.05 (1.29 to 3.26)**	0.003	**1.18 (1.03 to 1.35)**

Bolded values are statistically significant, p<0.05.

*Incidence rate is unadjusted and per 1000 person-years.

†Adjusted HRs per models as follows:

Model 1 adjusted for age, sex and race/ ethnicity.

Model 2: model 1 plus education, BMI and log (physical activity).

Model 3: model 2 plus smoking status, diabetes, eGFR, AV nodal blocker use, anticoagulant use and aspirin use.

Model 4: model 3 plus log (hsCRP), log (IL-6), log (fibrinogen) and log (D-dimer).

AV, atrioventricular; BMI, body mass index; bpm, beats per minute; eGFR, estimated glomerular filtration rate; hsCRP, high-sensitivity C reactive protein; IL-6, interleukin-6; MET, metabolic equivalent of task.

Sensitivity analysis among only the participants who were not taking AV-nodal blocker medications at baseline showed a higher risk of incident VTE for RHR 70–79 bpm and ≥80 bpm versus RHR ≤60 bpm, compared with primary analysis, with HRs of 1.56 (1.06 to 2.31) and 2.13 (1.30 to 3.48), respectively, in our fully adjusted model (model 4, table 3).

**Table 3:** Risk of incident venous thromboembolism associated with resting heart rate excluding participants on AV-nodal blockers: the Multi-Ethnic Study of Atherosclerosis), 2000–2015

Resting heart rate	≤60 bpm	60–69 bpm	70–79 bpm	≥80 bpm	P for trend	Per 10 bpm increment
N	2011	2252	1055	332	–	5650
VTE; n (%)	62 (3.1)	63 (2.8)	50 (4.7)	24 (7.2)	–	199 (3.5)
Person-years	25 499	28 414	12 937	3909	–	70 760
Incidence rate (95% CI)*	2.43 (1.90 to 3.12)	2.22 (1.73 to 2.84)	3.86 (2.93 to 5.10)	6.14 (4.11 to 9.16)	–	2.81 (2.45 to 3.23)
HRs (95% CI)†						
Model 1	1 (reference)	0.95 (0.67 to 1.35)	**1.69 (1.16 to 2.46**)	**2.60 (1.62 to 4.18)**	<0.001	**1.32 (1.15 to 1.52)**
Model 2	1 (reference)	0.90 (0.63 to 1.28)	**1.48 (1.01 to 2.16**)	**2.01 (1.24 to 3.27)**	0.002	**1.22 (1.06 to 1.41)**
Model 3	1 (reference)	0.92 (0.64 to 1.31)	**1.51 (1.03 to 2.22**)	**2.14 (1.31 to 3.49)**	0.001	**1.24 (1.07 to 1.44)**
Model 4	1 (reference)	0.95 (0.67 to 1.36)	**1.56 (1.06 to 2.31**)	**2.13 (1.30 to 3.48)**	0.001	**1.25 (1.08 to 1.45)**

Bolded values are statistically significant, P<0.05.

*Incidence rate is unadjusted and per 1000 person-years.

†Adjusted HRs per Models as follows:

Model 1 is adjusted for age, sex and race/ ethnicity.

Model 2: model 1 plus education, BMI and log (physical activity).

Model 3: model 2 plus smoking status, diabetes, eGFR, AV nodal blocker use, anticoagulant use and aspirin use.

Model 4: model 3 plus log (hsCRP), log (IL-6), log (fibrinogen) and log (D-dimer).

AV, atrioventricular; BMI, body mass index; bpm, beats per minute; eGFR, estimated glomerular filtration rate; hsCRP, high-sensitivity C reactive protein; IL-6, interleukin-6; MESA, Multi-Ethnic Study of Atherosclerosis; MET, metabolic equivalent of task; VTE, venous thromboembolism.

The mean RHR was also noted to be consistently higher in participants who developed VTE across the MESA follow-up visits compared with those who did not develop VTE (online supplementary table 4). However, there was no significant change in mean heart rate across time in participants who developed VTE (p=0.14) as compared with the trend seen among those who did not develop VTE (p<0.001). In sensitivity analyses, when RHR was modelled as a time-varying covariate updated at each of the MESA follow-up visits, results were similar to our primary models (online supplementary table 5).

**Figure 1:**
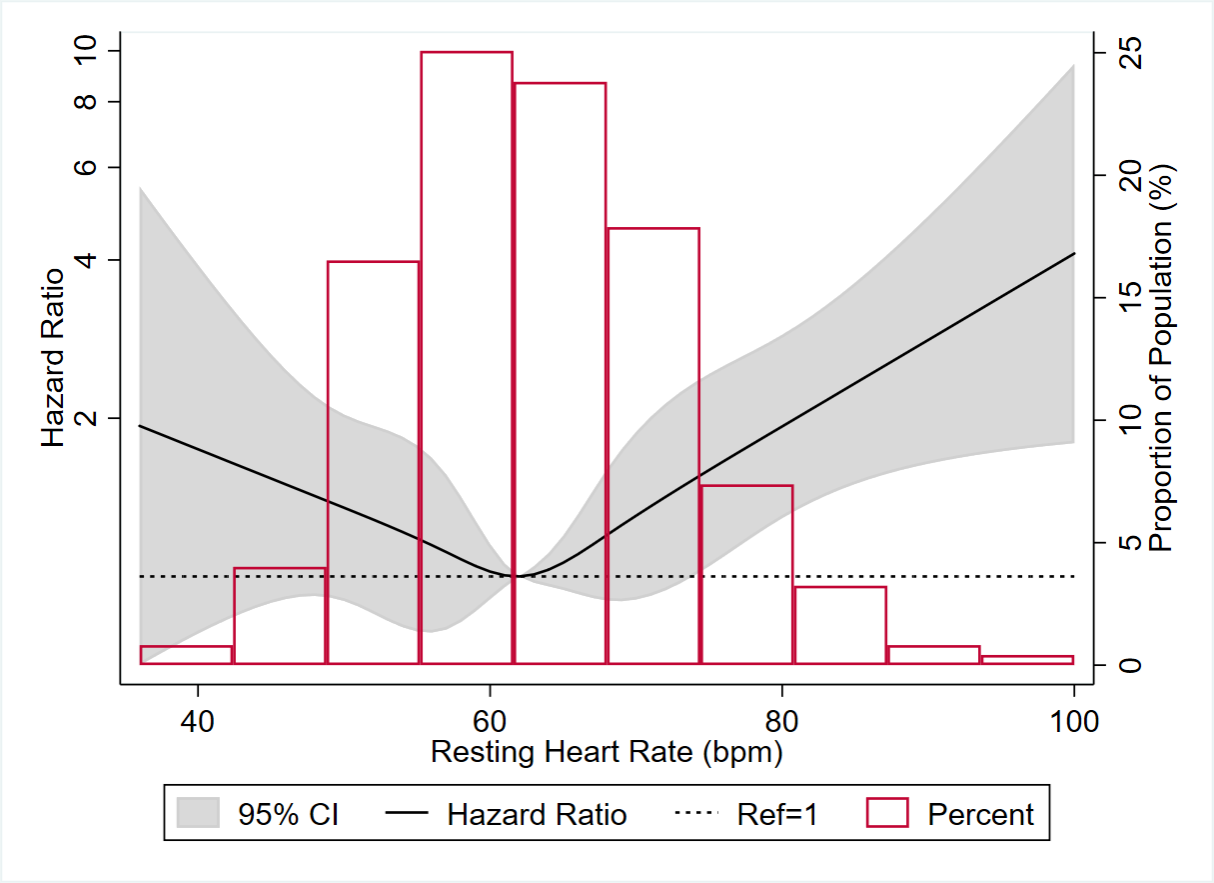
Restricted cubic spline* showing association of resting heart rate with incident venous thromboembolism in MESA, 2000–2015 *High extreme values of RHR (>100 bpm) were excluded (n=4) from graphical display. The histogram shows the distribution (proportion) of participants by RHR. The restricted cubic spline shows the HRs (95% CI) for the risk of incident VTE associated with RHR (continuous). The spline was generated using a Cox proportional hazards model, with median RHR (62 bpm) as reference, and with knots at 5th, 27.5th, 50th, 72.5th and 95th percentiles. The dotted line represents an HRof 1 (ref). The model was adjusted for age (continuous), sex (male;female), race/ ethnicity (white; black; Hispanic; Chinese), education (<high school; high school or vocational school; college, graduate or professional school), BMI (continuous) and physical activity level (metabolic equivalent-minutes /week of moderate or vigorous activity; log-transformed continuous). MESA, Multi-Ethnic Study of Atherosclerosis; MET, metabolic equivalent of task; RHR, resting heart rate; VTE, venous thromboembolism.

We also visually depicted the association using a restricted cubic spline model (figure 1), adjusted for model 2 covariates. The association appeared to be U-shaped, with decreased VTE risk for RHR less than the median value of 62 bpm (although with wide CIs) but increased VTE risk associated with elevated RHR when RHR>median.

Among participants who had data for RHR and inflammatory/coagulation factors (table 4), we found RHR to be significantly and positively associated with a number of inflammatory/coagulation factors after adjusting for age, sex and race/ethnicity, including hsCRP, IL-6, fibrinogen, D-dimer, VWF, Factor VIII, PAP, PAI-1, E-selectin and ICAM-1. RHR was not significantly associated with STM.

**Table 4:** Partial correlations of resting heart rate with inflammatory/coagulation factors: the Multi-Ethnic Study of Atherosclerosis, 2000–2002*

Marker	Whole cohort		Males		Females	
	N	r (p value)	N	r (p value)	N	r (p value)
hs-CRP	6714	0.15 (p<0.001)	3173	0.18 (p<0.001)	3541	0.12 (p<0.001)
Interleukin-6	6576	0.15 (p<0.001)	3101	0.17 (p<0.001)	3475	0.13 (p<0.001)
Fibrinogen	6721	0.15 (p<0.001)	3181	0.19 (p<0.001)	3540	0.13 (p<0.001)
D-dimer	6721	0.03 (p=0.005)	3182	0.04 (p=0.02)	3539	0.03 (p=0.12)
VWF	992	0.10 (p=0.002)	425	0.07 (p=0.14)	567	0.12 (p=0.01)
Factor VIII	6717	0.12 (p<0.001)	3179	0.11 (p<0.001)	3538	0.12 (p<0.001)
PAP	6584	0.05 (p<0.001)	3119	0.08 (p<0.001)	3465	0.002 (p=0.91)
PAI-1	968	0.13 (p<0.001)	418	0.16 (p=0.002)	550	0.11 (p=0.01)
STM	992	0.03 (p=0.39)	426	−0.01 (p=0.77)	566	0.08 (p=0.07)
E-selectin	994	0.16 (p<0.001)	426	0.21 (p<0.001)	568	0.11 (p=0.01)
ICAM-1	2610	0.07 (p<0.001)	1146	0.07 (p=0.03)	1464	0.07 (p=0.01)

r, Spearman’s correlation.

*Adjusted for age, sex (except in sex stratified analysis), and race/ethnicity.

hsCRP, high sensitivity C reactive protein; ICAM-1, Intercellular adhesion molecule 1; PAI-1, plasminogen activator inhibitor-1; PAP, plasmin–antiplasmin; STM, soluble thrombomodulin; VWF, von Willebrand factor.

## Discussion

In a large diverse community cohort of individuals free of VTE, CVD, atrial fibrillation or active cancer at baseline, we demonstrated that RHR was significantly associated with the incidence of VTE independent of several demographics, lifestyle and inflammatory factors. There was no interaction by sex or age, and the association appeared stronger after excluding participants on AV-nodal blocker medications. We also demonstrated that RHR had a significant and positive correlation, though weak, with several inflammatory and coagulation factors cross-sectionally including hsCRP, IL-6, fibrinogen, D-dimer, VWF, FVIII, PAP, PAI-1, E-selectin and ICAM-1.

RHR is a vital sign that is routinely measured at most clinical encounters, as well as self-monitored by individuals using fitness trackers and mobile devices. Prior studies have demonstrated that elevated RHR is associated with an increased risk of CVD, all-cause mortality,^6–10^ valvular heart disease^25 26^ and cognitive decline.^24^ However, there is little literature available on its role with VTE.

There are several plausible biological mechanisms by which an elevated RHR may contribute to VTE. Prior studies have shown that elevated RHR is positively associated with concentrations of inflammatory and coagulation markers,^7 11 13^ as our present study also demonstrates, and this may provide some explanation as to the role of elevated RHR in predicting VTE risk. In a study by Whelton *et al*, which included participants enrolled in the MESA study, it was shown that elevated RHR was associated with higher levels of hs-CRP, IL-6 and fibrinogen.^11^ In another study in the Danish population, Jensen *et al* demonstrated that RHR was associated with hsCRP and fibrinogen.^7^ RHR may also be associated with activation of haemostatic and thrombotic factors. In a pilot study of participants with mitral stenosis and atrial fibrillation, participants with higher heart rates (>100 bpm) were found to have significantly higher levels of coagulation factors (prothrombin fragment 1+2, thrombin antithrombin III and PAI) when compared with participants with lower heart rates (≤100 bpm).^14^

RHR is thought to reflect a balance of sympathetic and parasympathetic nervous systems. The parasympathetic system predominates in resting states, so an elevated RHR may be reflective of decreased parasympathetic tone and increased sympathetic stimulation. Alteration in the sympathetic nervous system may predispose to thrombosis risk,^27 28^ which may be another mechanism linking RHR to VTE formation. At rest, individuals with increased physical fitness tend to have higher parasympathetic tone and better autonomic function, which contributes to a lower RHR.^9 11^ However, a prior study found that there remained an association of RHR with mortality risk even after adjusting for measured fitness (METS on treadmill testing).^10^ RHR has also been associated with progression of valvular calcification and stenosis, which may be due to mechanical shear stress from enhanced cardiac output.^25 26^

In sum, our findings re-enforce the association of RHR with inflammatory and coagulation processes. In addition, we now newly show an association of RHR with future VTE risk, independent of several traditional VTE and inflammatory risk factors. This suggests that the risk of RHR with VTE may not fully be explained by its association with inflammatory markers and may be due to other causes. However, it is important to note that the inflammatory markers assessed in this study were measured only once at baseline, and adjustment for baseline values may not capture the long-term exposure of these markers or their intraindividual variability. More work is needed to be done to establish these mechanisms. However, to confirm that the associations we found were not spurious, after completing the above analysis in MESA, we then sought out to confirm whether RHR was associated with VTE in another large prospective cohort, the ARIC study, which notably had adjudicated VTE outcomes. This confirmatory work from our group was recently published and similar associations were found for RHR and incident VTE, with HRs (95% CI) of 1.44 (1.01 to 2.06) comparing RHR of ≥80 bpm to <60 bpm and 1.11 (1.02 to 1.21) per 10 bpm increment in RHR.^29^

Our study has many strengths including the prospective design and utilisation of data from the well-characterised MESA cohort, which allowed us to rigorously adjust for numerous potentially confounding demographic, lifestyle and VTE risk factors. We studied a large sample of racially/ethnically diverse men and women over a prolonged time course of 14 years to study the long-term associations between baseline RHR (and time-varying RHR) and incident VTE. Nevertheless, our findings have some limitations, which are worth noting. First, cases of VTE were identified from medical records using ICD codes and were not adjudicated. This may have led to some false positives or negatives. However, as noted above, our confirmatory follow-up analysis in the ARIC cohort, which did have validated VTE outcomes, found similar results.^29^ Second, we made use of RHR at baseline for our primary analysis, which may not be representation of RHR during follow-up. Nevertheless, our sensitivity analysis using RHR at multiple follow-up visits still showed significant associations between elevated RHR and VTE risk. Third, the observational design does limit causal inference. Although we attempted to adjust for multiple VTE risk factors, residual confounding may still exist due to the multifactorial nature and complex pathophysiology of VTE. For example, elevated RHR may be a manifestation for someone in a poorer health state.

## Conclusions

In summary, in a large multiethnic community-based cohort, we have demonstrated that elevated RHR was associated with an increased risk of incident VTE and with an increased level of several inflammatory and coagulation factors. Mechanisms for this association may be activation of inflammatory pathway, sympathetic nervous system and mechanical shear stress. However, further studies are needed to determine whether the associations of RHR and VTE are causal or secondary to another underlying process. RHR, which is an easily assessed clinical variable, is underused for vascular risk prediction. Whether the modification of RHR by lifestyle or pharmacological intervention would reduce the future risk of VTE is unknown and warrants further exploration.
