# Supplementary material for: Resting heart rate and incident venous thromboembolism: the Multi-Ethnic Study of Atherosclerosis
Source: Open Heart. 2020 Feb 20;7(1):e001080. doi: 10.1136/openhrt-2019-001080 (PMC7046973; doi:10.1136/openhrt-2019-001080)
Supplement: Supplementary data [file openhrt-2019-001080supp001.pdf]

Supplemental Figure 1. Participant Flow Chart, the Multi-Ethnic Study of Atherosclerosis (MESA)

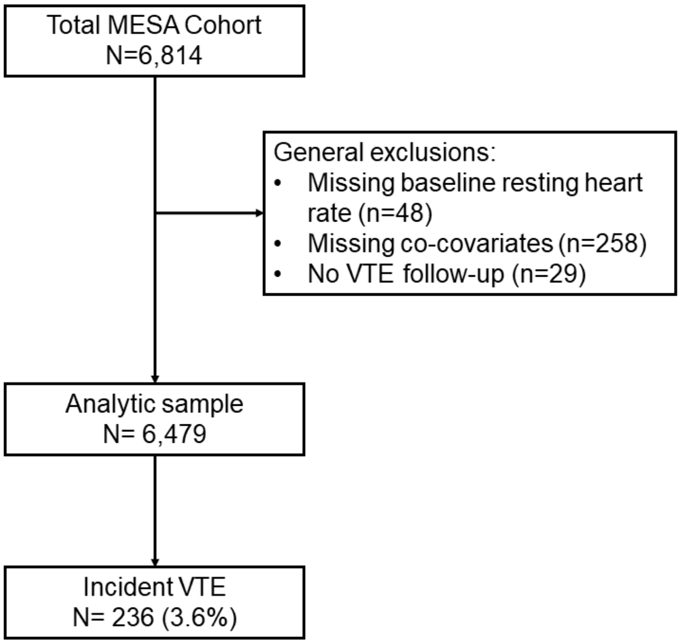

VTE=venous thromboembolism

Awotoye J, *et al. Open Heart* 2020; 7:e001080. doi: 10.1136/openhrt-2019-001080

Supplemental Table 1. ICD-9 and ICD-10-CM codes used to define VTE endpoint, MESA

ICD-9-CM CODES USED TO DEFINE VTE ENDPOINT

ICD-9 Code	Code Description
415.	Acute pulmonary heart disease
415.1	Pulmonary embolism and infarction
415.11	Iatrogenic pulmonary embolism and infarction
415.12	Septic pulmonary embolism
415.13	Saddle embolus of pulmonary artery
415.19	Other pulmonary embolism and infarction
451.	Phlebitis and thrombophlebitis
451.1	Phlebitis and thrombophlebitis of deep veins of lower extremities
451.11	Phlebitis and thrombophlebitis of femoral vein (deep) (superficial)
451.19	Phlebitis and thrombophlebitis of deep veins of lower extremities, other
451.2	Phlebitis and thrombophlebitis of lower extremities, unspecified
451.81	Phlebitis and thrombophlebitis of iliac vein
451.9	Phlebitis and thrombophlebitis of unspecified site
453.1	Thrombophlebitis migrans
453.2	Other venous embolism and thrombosis of inferior vena cava
453.4	Venous embolism and thrombosis of deep vessels of lower extremity
453.40	Acute venous embolism and thrombosis of unspecified deep vessels of lower extremity
453.41	Acute venous embolism and thrombosis of deep vessels of proximal lower extremity
453.42	Acute venous embolism and thrombosis of deep vessels of distal lower extremity
453.5	Chronic venous embolism and thrombosis of unspecified deep vessels of lower extremity

Awotoye J, *et al. Open Heart* 2020; 7:e001080. doi: 10.1136/openhrt-2019-001080

ICD-9 Code	Code Description
453.50	Chronic venous embolism and thrombosis of unspecified deep vessels of lower extremity
453.51	Chronic venous embolism and thrombosis of deep vessels of proximal lower extremity
453.52	Chronic venous embolism and thrombosis of deep vessels of distal lower extremity
453.8	Other venous embolism and thrombosis of other specified veins
453.82	Acute venous embolism and thrombosis of deep veins of upper extremity
453.9	Other venous embolism and thrombosis of unspecified site
415.	Acute pulmonary heart disease
415.1	Pulmonary embolism and infarction
415.11	Iatrogenic pulmonary embolism and infarction
415.12	Septic pulmonary embolism
415.13	Saddle embolus of pulmonary artery
415.19	Other pulmonary embolism and infarction
451.	Phlebitis and thrombophlebitis
451.1	Phlebitis and thrombophlebitis of deep veins of lower extremities
451.11	Phlebitis and thrombophlebitis of femoral vein (deep) (superficial)
451.19	Phlebitis and thrombophlebitis of deep veins of lower extremities, other
451.2	Phlebitis and thrombophlebitis of lower extremities, unspecified
451.81	Phlebitis and thrombophlebitis of iliac vein
451.9	Phlebitis and thrombophlebitis of unspecified site
453.1	Thrombophlebitis migrans
453.2	Other venous embolism and thrombosis of inferior vena cava
453.4	Venous embolism and thrombosis of deep vessels of lower extremity
453.40	Acute venous embolism and thrombosis of unspecified deep vessels of lower extremity

Awotoye J, *et al. Open Heart* 2020; 7:e001080. doi: 10.1136/openhrt-2019-001080

ICD-9 Code	Code Description
453.41	Acute venous embolism and thrombosis of deep vessels of proximal lower extremity
453.42	Acute venous embolism and thrombosis of deep vessels of distal lower extremity
453.5	Chronic venous embolism and thrombosis of unspecified deep vessels of lower extremity
453.50	Chronic venous embolism and thrombosis of unspecified deep vessels of lower extremity
453.51	Chronic venous embolism and thrombosis of deep vessels of proximal lower extremity
453.52	Chronic venous embolism and thrombosis of deep vessels of distal lower extremity
453.8	Other venous embolism and thrombosis of other specified veins
453.82	Acute venous embolism and thrombosis of deep veins of upper extremity
453.9	Other venous embolism and thrombosis of unspecified site

Awotoye J, *et al. Open Heart* 2020; 7:e001080. doi: 10.1136/openhrt-2019-001080

ICD-10-CM CODES USED TO DEFINE VTE ENDPOINT

ICD-10 Code	Code Description
I26.	Pulmonary embolism
I80.1	Phlebitis and thrombophlebitis of femoral vein
I80.10	Phlebitis and thrombophlebitis of unspecified femoral vein
I80.11	Phlebitis and thrombophlebitis of right femoral vein
I80.12	Phlebitis and thrombophlebitis of left femoral vein
I80.13	Phlebitis and thrombophlebitis of femoral vein, bilateral
I80.2	Phlebitis and thrombophlebitis of other and unspecified deep vessels of lower extremities
I80.20	Phlebitis and thrombophlebitis of unspecified deep vessels of lower extremities
I80.201	Phlebitis and thrombophlebitis of unspecified deep vessels of right lower extremity
I80.202	Phlebitis and thrombophlebitis of unspecified deep vessels of left lower extremity
I80.203	Phlebitis and thrombophlebitis of unspecified deep vessels of lower extremities, bilateral
I80.209	Phlebitis and thrombophlebitis of unspecified deep vessels of unspecified lower extremity
I80.21	Phlebitis and thrombophlebitis of iliac vein
I80.211	Phlebitis and thrombophlebitis of right iliac vein
I80.212	Phlebitis and thrombophlebitis of left iliac vein
I80.213	Phlebitis and thrombophlebitis of iliac vein, bilateral
I80.219	Phlebitis and thrombophlebitis of unspecified iliac vein
I80.22	Phlebitis and thrombophlebitis of popliteal vein
I80.221	Phlebitis and thrombophlebitis of right popliteal vein
I80.222	Phlebitis and thrombophlebitis of left popliteal vein
I80.223	Phlebitis and thrombophlebitis of popliteal vein, bilateral

Awotoye J, *et al. Open Heart* 2020; 7:e001080. doi: 10.1136/openhrt-2019-001080

ICD-10 Code	Code Description
I80.229	Phlebitis and thrombophlebitis of unspecified popliteal vein
I80.23	Phlebitis and thrombophlebitis of tibial vein
I80.231	Phlebitis and thrombophlebitis of right tibial vein
I80.232	Phlebitis and thrombophlebitis of left tibial vein
I80.233	Phlebitis and thrombophlebitis of tibial vein, bilateral
I80.239	Phlebitis and thrombophlebitis of unspecified tibial vein
I80.29	Phlebitis and thrombophlebitis of other deep vessels of lower extremities
I80.291	Phlebitis and thrombophlebitis of other deep vessels of right lower extremity
I80.292	Phlebitis and thrombophlebitis of other deep vessels of left lower extremity
I80.293	Phlebitis and thrombophlebitis of other deep vessels of lower extremity, bilateral
I80.299	Phlebitis and thrombophlebitis of other deep vessels of unspecified lower extremity
I80.3	Phlebitis and thrombophlebitis of lower extremities, unspecified
I80.8	Phlebitis and thrombophlebitis of other sites
I80.9	Phlebitis and thrombophlebitis of unspecified site
I82.1	Thrombophlebitis migrans
I82.22	Embolism and thrombosis of inferior vena cava
I82.4	Acute embolism and thrombosis of deep veins of lower extremity
I82.40	Acute embolism and thrombosis of unspecified deep veins of lower extremity
I82.401	Acute embolism and thrombosis of unspecified deep veins of right lower extremity
I82.402	Acute embolism and thrombosis of unspecified deep veins of left lower extremity
I82.403	Acute embolism and thrombosis of unspecified deep veins of lower extremity, bilateral
I82.409	Acute embolism and thrombosis of unspecified deep veins of unspecified lower extremity
I82.41	Acute embolism and thrombosis of femoral vein

ICD-10 Code	Code Description
I82.411	Acute embolism and thrombosis of right femoral vein
I82.412	Acute embolism and thrombosis of left femoral vein
I82.413	Acute embolism and thrombosis of femoral vein, bilateral
I82.419	Acute embolism and thrombosis of unspecified femoral vein
I82.42	Acute embolism and thrombosis of iliac vein
I82.421	Acute embolism and thrombosis of right iliac vein
I82.422	Acute embolism and thrombosis of left iliac vein
I82.423	Acute embolism and thrombosis of iliac vein, bilateral
I82.429	Acute embolism and thrombosis of unspecified iliac vein
I82.43	Acute embolism and thrombosis of popliteal vein
I82.431	Acute embolism and thrombosis of right popliteal vein
I82.432	Acute embolism and thrombosis of left popliteal vein
I82.433	Acute embolism and thrombosis of popliteal vein, bilateral
I82.439	Acute embolism and thrombosis of unspecified popliteal vein
I82.44	Acute embolism and thrombosis of tibial vein
I82.441	Acute embolism and thrombosis of right tibial vein
I82.442	Acute embolism and thrombosis of left tibial vein
I82.443	Acute embolism and thrombosis of tibial vein, bilateral
I82.449	Acute embolism and thrombosis of unspecified tibial vein
I82.49	Acute embolism and thrombosis of other specified deep vein of lower extremity
I82.491	Acute embolism and thrombosis of other specified deep vein of right lower extremity
I82.492	Acute embolism and thrombosis of other specified deep vein of left lower extremity
I82.493	Acute embolism and thrombosis of other specified deep vein of lower extremity, bilateral

ICD-10 Code	Code Description
I82.499	Acute embolism and thrombosis of other specified deep vein of unspecified lower extremity
I82.4Y	Acute embolism and thrombosis of unspecified deep veins of proximal lower extremity
I82.4Y1	Acute embolism and thrombosis of unspecified deep veins of right proximal lower extremity
I82.4Y2	Acute embolism and thrombosis of unspecified deep veins of left proximal lower extremity
I82.4Y3	Acute embolism and thrombosis of unspecified deep veins of proximal lower extremity, bilateral
I82.4Y9	Acute embolism and thrombosis of unspecified deep veins of unspecified proximal lower extremity
I82.4Z	Acute embolism and thrombosis of unspecified deep veins of distal lower extremity
I82.4Z1	Acute embolism and thrombosis of unspecified deep veins of right distal lower extremity
I82.4Z2	Acute embolism and thrombosis of unspecified deep veins of left distal lower extremity
I82.4Z3	Acute embolism and thrombosis of unspecified deep veins of distal lower extremity, bilateral
I82.4Z9	Acute embolism and thrombosis of unspecified deep veins of unspecified distal lower extremity
I82.5	Chronic embolism and thrombosis of deep veins of lower extremity
I82.50	Chronic embolism and thrombosis of unspecified deep veins of lower extremity
I82.501	Chronic embolism and thrombosis of unspecified deep veins of right lower extremity
I82.502	Chronic embolism and thrombosis of unspecified deep veins of left lower extremity
I82.503	Chronic embolism and thrombosis of unspecified deep veins of lower extremity, bilateral
I82.509	Chronic embolism and thrombosis of unspecified deep veins of unspecified lower extremity
I82.51	Chronic embolism and thrombosis of femoral vein
I82.511	Chronic embolism and thrombosis of right femoral vein
I82.512	Chronic embolism and thrombosis of left femoral vein
I82.513	Chronic embolism and thrombosis of femoral vein, bilateral
I82.519	Chronic embolism and thrombosis of unspecified femoral vein
I82.52	Chronic embolism and thrombosis of iliac vein

Awotoye J, *et al. Open Heart* 2020; 7:e001080. doi: 10.1136/openhrt-2019-001080

ICD-10 Code	Code Description
I82.521	Chronic embolism and thrombosis of right iliac vein
I82.522	Chronic embolism and thrombosis of left iliac vein
I82.523	Chronic embolism and thrombosis of iliac vein, bilateral
I82.529	Chronic embolism and thrombosis of unspecified iliac vein
I82.53	Chronic embolism and thrombosis of popliteal vein
I82.531	Chronic embolism and thrombosis of right popliteal vein
I82.532	Chronic embolism and thrombosis of left popliteal vein
I82.533	Chronic embolism and thrombosis of popliteal vein, bilateral
I82.539	Chronic embolism and thrombosis of unspecified popliteal vein
I82.54	Chronic embolism and thrombosis of tibial vein
I82.541	Chronic embolism and thrombosis of right tibial vein
I82.542	Chronic embolism and thrombosis of left tibial vein
I82.543	Chronic embolism and thrombosis of tibial vein, bilateral
I82.549	Chronic embolism and thrombosis of unspecified tibial vein
I82.59	Chronic embolism and thrombosis of other specified deep vein of lower extremity
I82.591	Chronic embolism and thrombosis of other specified deep vein of right lower extremity
I82.592	Chronic embolism and thrombosis of other specified deep vein of left lower extremity
I82.593	Chronic embolism and thrombosis of other specified deep vein of lower extremity, bilateral
I82.599	Chronic embolism and thrombosis of other specified deep vein of unspecified lower extremity
I82.5Y	Chronic embolism and thrombosis of unspecified deep veins of proximal lower extremity
I82.5Y1	Chronic embolism and thrombosis of unspecified deep veins of right proximal lower extremity
I82.5Y2	Chronic embolism and thrombosis of unspecified deep veins of left proximal lower extremity
I82.5Y3	Chronic embolism and thrombosis of unspecified deep veins of proximal lower extremity, bilateral

Awotoye J, *et al. Open Heart* 2020; 7:e001080. doi: 10.1136/openhrt-2019-001080

ICD-10 Code	Code Description
I82.5Y9	Chronic embolism and thrombosis of unspecified deep veins of unspecified proximal lower extremity
I82.5Z	Chronic embolism and thrombosis of unspecified deep veins of distal lower extremity
I82.5Z1	Chronic embolism and thrombosis of unspecified deep veins of right distal lower extremity
I82.5Z2	Chronic embolism and thrombosis of unspecified deep veins of left distal lower extremity
I82.5Z3	Chronic embolism and thrombosis of unspecified deep veins of distal lower extremity, bilateral
I82.5Z9	Chronic embolism and thrombosis of unspecified deep veins of unspecified distal lower extremity
I82.9	Embolism and thrombosis of unspecified vein
I82.90	Acute embolism and thrombosis of unspecified vein
I82.91	Chronic embolism and thrombosis of unspecified vein

Awotoye J, *et al. Open Heart* 2020; 7:e001080. doi: 10.1136/openhrt-2019-001080

Supplemental Table 2: Baseline characteristics of study participants stratified by resting heart rate categories, MESA, 2000-2002

Resting heart rate	≤60 bpm	60 - 69 bpm	70 - 79 bpm	≥80 bpm	p-value
N	2434	2540	1143	362	-
Age, years	62.5 ± 10.3	61.8 ± 10.2	62 ± 10.4	62.4 ± 10.1	0.10
Female	1121 (46.1%)	1429 (56.3%)	657 (57.5%)	216 (59.7%)	<0.001
Race					0.01
White	957 (39.3%)	981 (38.6%)	435 (38.1%)	131 (36.2%)	-
Black	700 (28.8%)	638 (25.1%)	302 (26.4%)	117 (32.3%)	-
Hispanic	508 (20.9%)	574 (22.6%)	270 (23.6%)	79 (21.8%)	-
Chinese	269 (11.1%)	347 (13.7%)	136 (11.9%)	35 (9.7%)	-
Education					0.001
Less than high school	402 (16.5%)	476 (18.7%)	229 (20.0%)	69 (19.1%)	-
High school or vocational school	986 (40.5%)	1041 (41%)	482 (42.2%)	174 (48.1%)	-
College, graduate or professional school	1046 (43.0%)	1023 (40.3%)	432 (37.8%)	119 (32.9%)	-
Smoking status					0.01
Never	1167 (48.0%)	1297 (51.1%)	609 (53.3%)	195 (53.9%)	-
Former	966 (39.7%)	915 (36.0%)	381 (33.3%)	119 (32.9%)	-
Current	301 (12.4%)	328 (12.9%)	153 (13.4%)	48 (13.3%)	-
BMI, kg/m^2	27.7 ± 5	28.2 ± 5.5	29.1 ± 5.7	30.4 ± 6.6	<0.001
Physical activity, MET-minutes/week*	4320 (2160 - 8250)	3930 (1980 - 7429)	3758 (1778 - 7080)	3094 (1380 - 5723)	<0.001
Diabetes	196 (8.1%)	296 (11.7%)	217 (19.0%)	105 (29.0%)	<0.001
eGFR, ml/min per 1.73 m^2	76.4 ± 15.7	78.4 ± 15.9	78.8 ± 16.9	78.6 ± 18.6	<0.001
AV nodal blocker medication	423 (17.4%)	288 (11.3%)	88 (7.7%)	30 (8.3%)	<0.001
Anticoagulant use	12 (0.5%)	10 (0.4%)	1 (0.1%)	1 (0.3%)	0.31
Aspirin use	639 (26.3%)	632 (24.9%)	265 (23.2%)	95 (26.2%)	0.24
hsCRP, mg/L*	1.5 (0.7 - 3.4)	2.0 (0.9 - 4.3)	2.5 (1.0 - 5.0)	2.9 (1.3 - 6.2)	<0.001
Interleukin-6, pg/mL*	1.1 (0.7 - 1.7)	1.2 (0.8 - 1.9)	1.3 (0.9 - 2.1)	1.5 (1.0 - 2.5)	<0.001
Fibrinogen, mg/dL*	329 (290 - 376)	338 (293 - 388)	352 (307 - 406)	367 (317 - 419)	<0.001
D-dimer, mcg/mL*	0.2 (0.1 - 0.4)	0.2 (0.1 - 0.4)	0.2 (0.1 - 0.4)	0.3 (0.2 - 0.4)	0.02

p-values derived from one way ANOVA and Kruskal-Wallis for continuous variables and Chi-square test for categorical variables.

Awotoye J, *et al. Open Heart* 2020; 7:e001080. doi: 10.1136/openhrt-2019-001080

Supplemental Table 3. Hazard ratios* (95% confidence interval) of incident venous thromboembolism associated with resting heart rate, stratified by age groups, 2000-2015, the Multi-Ethnic Study of Atherosclerosis (MESA)

Age	44-53 years			54-63 years			64-73 years			74-84 years		
Heart rate (bpm)	VTE, n (%)	N	Hazard ratio*	VTE, n (%)	N	Hazard ratio*	VTE, n (%)	N	Hazard ratio*	VTE, n (%)	N	Hazard ratio*
<60	8 (1.32%)	605	1 (reference)	25 (3.88%)	644	1 (reference)	27 (3.52%)	766	1 (reference)	23 (5.49%)	419	1 (reference)
60-69	4 (0.6%)	662	0.49 (0.14, 1.66)	23 (3.1%)	741	0.79 (0.44, 1.39)	24 (3.27%)	735	0.93 (0.54, 1.63)	24 (5.97%)	402	1.04 (0.58, 1.84)
70-79	4 (1.35%)	297	0.96 (0.27, 3.33)	12 (3.73%)	322	1.01 (0.51, 2.03)	20 (5.83%)	343	1.53 (0.84, 2.78)	16 (8.84%)	181	1.69 (0.88, 3.24)
>80	5 (5.62%)	89	**4.86 (1.37, 17.28)**	5 (4.46%)	112	1.12 (0.42, 3.01)	7 (7%)	100	1.76 (0.75, 4.1)	9 (14.75%)	61	**3.17 (1.43, 7.05)**
Total/ p-for-trend	21 (1.27%)	1,653	0.07	65 (3.57%)	1,819	0.91	78 (4.01%)	1,944	0.10	72 (6.77%)	1,063	0.01
Hazard ratio* per 10 bpm increment	1.38 (0.86, 2.21)			1.02 (0.78, 1.34)			1.16 (0.92, 1.47)			**1.30 (1.02, 1.64)**		

VTE = venous thromboembolism

* Adjusted for sex, and race/ ethnicity, education, BMI, and physical activity

† p for interaction by age for categorical heart rate was 0.64 and for continuous heart rate was 0.55

Awotoye J, *et al. Open Heart* 2020; 7:e001080. doi: 10.1136/openhrt-2019-001080

Supplemental Table 4. Mean resting heart rate at each visit by incident VTE status: the Multi-Ethnic Study of Atherosclerosis (MESA)

Mean heart rate (bpm)	MESA Exam					p-value*
	Exam 1	Exam 2	Exam 3	Exam 4	Exam 5	
No VTE	63.0	65.5	64.9	64.8	64.3	<0.001
VTE	64.6	68.3	66.7	65.8	65.6	0.14

VTE = venous thromboembolism

*p-value is derived from a linear-mixed effects model with random intercepts and slopes

Supplemental Table 5. Hazard ratios (95% confidence interval) of incident venous thromboembolism associated with time varying† resting heart rate, 2000-2015, the Multi-Ethnic Study of Atherosclerosis (MESA)

Resting heart rate	≤60 bpm	60 - 69 bpm	70 - 79 bpm	≥80 bpm	p-for-trend	Per 10 bpm increment
Incidence rate** (95% CI)	2.52 (1.99 - 3.2)	2.49 (1.99 - 3.11)	3.37 (2.6 - 4.38)	5.71 (4.1 - 7.95)	-	2.93 (2.58 - 3.33)
Model 1	1 (reference)	1.14 (0.82 - 1.58)	**1.55 (1.09 - 2.21)**	**2.28 (1.52 - 3.43)**	<0.001	**1.26 (1.13 - 1.41)**
Model 2	1 (reference)	1.15 (0.83 - 1.59)	**1.44 (1.01 - 2.06)**	**2.12 (1.41 - 3.19)**	<0.001	**1.23 (1.10 - 1.37)**
Model 3	1 (reference)	1.12 (0.80 - 1.56)	1.38 (0.96 - 1.99)	**2.04 (1.34 - 3.09)**	0.001	**1.19 (1.07 - 1.34)**
Model 4	1 (reference)	1.12 (0.80 - 1.56)	1.43 (0.99 - 2.06)	**2.04 (1.33 - 3.10)**	0.001	**1.20 (1.07 - 1.34)**

VTE = venous thromboembolism

† Time-varying meant these variables were updated at each MESA visit (MESA 1, 2, 3, 4, and 5).
* These variables were fixed at baseline.
**Incidence rate is unadjusted and per 1,000 person-years

Model 1 is adjusted for age*, sex*, and race/ ethnicity*
Model 2: model 1 plus education*, BMI†, and log (physical activity)†
Model 3: model 2 plus smoking status†, diabetes†, eGFR†, AV nodal blocker use†, anticoagulant use† and aspirin use†
Model 4: model 3 plus log (hsCRP)*, log (IL-6)*, log (fibrinogen)* and log (D-dimer)*

Awotoye J, *et al. Open Heart* 2020; 7:e001080. doi: 10.1136/openhrt-2019-001080
